# Impact of continuous nine-year application of biochar on potassium supplying capacity of the soil

**DOI:** 10.3389/fpls.2026.1866440

**Published:** 2026-06-24

**Authors:** Wanning Dai, Zhengrong Bao, Jun Meng, Zunqi Liu, Xiao Liang

**Affiliations:** 1 School of Agriculture, Liaodong University, Dandong, China; 2 Institute of Advanced Characteristic Agriculture Studies, Liaodong University, Dandong, China; 3 College of Agronomy, Shenyang Agricultural University, Shenyang, China; 4 Key Laboratory of Biochar and Soil Improvement, National Biochar Institute of Shenyang Agricultural University, Ministry of Agriculture and Rural Affairs, Shenyang, China

**Keywords:** biochar, K release kinetics, K supply potential, quantity/intensity, X-ray diffraction

## Abstract

**Introduction:**

Straw biochar can significantly increase the content of available potassium (K) in soil, whereas its regulatory effect on soil K supply capacity remains unclear and lacks long-term field verification. To clarify the response mechanism of soil K supply capacity to long-term straw biochar amendment, a 9-year continuous field experiment was carried out to explore the improvement effect of straw biochar on soil K supply characteristics and K uptake by maize.

**Methods:**

This study was conducted based on a long-term field experiment in Shenyang Agricultural University under a humid and sub-humid monsoon climate, with Haplic Luvisols as the tested soil. After 9 consecutive maize growing seasons, soil samples at the 0–20 cm depth were collected. A two-factor randomized block design was set up with four treatments, including B0K0 (no biochar and no K fertilizer), B0K1 (K fertilizer only), B1K0 (biochar only), and B1K1 (biochar with K fertilizer).

**Results:**

9 consecutive years of straw biochar application significantly increased illite content. Compared to B_0_K_0_, the illite proportion in B_1_K_0_ and B_1_K_1_ increased by 16.15% and 19.84%, respectively. The soil K quantity/intensity (Q/I) curve and K release kinetic curves showed that biochar significantly improved soil K supply pool (−ΔK_0_) and K supply intensity (ARe) by 60.76% and 36.06%. In addition, biochar reduced the content of exclusively adsorbed K (K_x_) and the free exchange energy of K (−ΔG), and effectively increased the cumulative release of soil K.

**Discussion:**

These variations induced by biochar application effectively alleviated the negative K balance of farmland soil, comprehensively improved soil K supply capacity, and facilitated K uptake and utilization of maize, which further promoted the accumulation of plant dry matter. This long-term field study confirms that the combined application of straw biochar and K fertilizer is an effective measure to improve soil K availability, which provides reliable scientific basis for K nutrient regulation in farmland soil.

## 1 Introduction

Potassium (K) is a vital nutrient for crop growth (Carciochi et al., 2025). Despite its abundance in the Earth’s crust (Chen et al., 2020), only a limited fraction is bioavailable to plants. Crops in unfertilized systems depend almost entirely on mineral weathering to obtain the K essential for their growth (Portela et al., 2019). However, in intensive cropping systems, the K released through mineral weathering fails to satisfy the elevated demands of high-yielding cultivars. When crop K removal persistently surpasses external K supplementation, soils inevitably develop a negative K balance. Prolonged K deficiencies accelerate the exhaustion of soil K stocks, resulting in severe deterioration of the soil’s ability to provide plant-available K (Sheldrick et al., 2003). Therefore, ensuring sufficient external K application is now recognized as a crucial agricultural practice for achieving both yield targets and long-term soil K sustainability. Currently, mineral K fertilizers serve as the predominant solution for mitigating soil K deficiencies in agricultural systems. Although highly efficient in the short term, such fertilizers carry significant disadvantages, particularly regarding nutrient leaching, high input costs, and potential environmental damage (Qiu et al., 2014; Han et al., 2021, 2023). Pyrolysis of straw into biochar for field application represents a key strategy for agricultural waste valorization, the resulting biochar, containing substantial K reserves, demonstrates remarkable potential for soil amelioration and nutrient recycling. Biochar plays the following key roles in the management of soil K: (1) direct replenishment of soil K reserves (Zhang et al., 2020b), (2) mitigation of K leaching (Feng et al., 2022), and (3) enhancement of both K bioavailability and overall soil quality (Lin et al., 2018). This integrated approach effectively supports the principles of sustainable agricultural intensification. Existing research has mainly focused on biochar’s impacts on total K content and immediate availability, while its effect on soil indigenous potassium supply capacity is still poorly documented. Given that soil potassium-supplying capacity serves as a critical indicator for sustaining long-term K fertility, this key property in biochar-amended soils therefore warrants in-depth investigation; exploring biochar’s influence on it is essential to accurately evaluate the material’s potential for long−term soil fertility improvement.

The NH_4_OAc extraction method is the most commonly used method for assessing soil K supply capacity. However, a major constraint of this method in long-term K management studies is its exclusive focus on exchangeable and water-soluble K, disregarding the substantial role of plant-available non-exchangeable K in sustaining crop K uptake (Bilias et al., 2023). The soil K quantity-intensity (Q/I) relationship provides an integrated assessment of critical K availability parameters, encompassing K intensity (solution concentration), K capacity (reservoir size), and K buffering capacity (resistance to concentration changes) (Islam et al., 2017; Bilias and Barbayiannis, 2019). Research has demonstrated that the Q/I curve can provide a more reliable assessment of soil K availability compared to exchangeable K measurements (Das et al., 2018). The Q/I relationship represents a comprehensive indicator of a soil’s long-term K supply capacity (Xiu et al., 2025) and has been successfully employed in previous studies to evaluate how chemical fertilizer and manure applications affect soil K-supplying potential (Rupa et al., 2003). However, despite its demonstrated utility, the application of Q/I analysis remains notably absent in studies investigating biochar’s impact on soil K supply dynamics, representing a critical gap in current research.

Clay minerals serve as crucial reservoirs and primary sources of potassium in soils, where their specific composition and abundance significantly influence both the soil’s K-supplying capacity and total K content (Zhang et al., 2016; Ndzana et al., 2019). The 2:1 clay minerals (including montmorillonite, vermiculite, and illite) play a particularly crucial role in K dynamics. Their expandable layered structure and high cation exchange capacity (CEC) endow these clay minerals with exceptional K retention and release capabilities. Empirical studies have demonstrated a positive correlation between soil illite content and potassium-supplying capacity, highlighting its critical role in sustaining long-term K availability (Zhang et al., 2020a).While the correlation between soil clay minerals and potassium (K) content is well-studied, the mechanism by which biochar could enhance K availability through modifying clay mineral composition and buffering capacity remains unexplored. Therefore, this study aimed to clarify the changes in soil K supply capacity after nine years of biochar amendment. We proposed three hypotheses: (1) long-term biochar application increases the relative abundance of soil illite; (2) biochar incorporation improves soil K-supplying capacity (Q) and K intensity (I), thus enhancing plant-available K; and (3) biochar amendment promotes cumulative soil K release and mitigates a negative soil K balance.

## 2 Materials and methods

### 2.1 Site description

A field study (2013 - 2021) was conducted at the experimental station of Shenyang Agricultural University, Shenyang, Liaoning Province (41°49′N, 123°33′E) was selected as the experimental site. The site was characterized by a temperate humid-semi humid monsoon climate, with an average annual temperature of about 7.9 °C and an annual rainfall of 574–684 mm. The soil type was classified as Haplic Luvisols (World Reference Base for Soil Resources classification), and the soil particles consisted of 16.7% sand, 24.9% clay, and 58.4% silt. The properties of the topsoil at the beginning of the experiment, determined according to Bao (2000) soil testing method, are shown in **Table 1**.

**Table 1:** Basic properties of soil and biochar use in this study.

Soil index	Biochar	Soil
pH	9.2	7.4
Organic matter (g·kg−1)	--	13.84
Total C (g kg−1)	660	11.0
Total N (g kg−1)	12.7	1.2
Total P (g kg−1)	8.87	0.38
Total K (g kg−1)	32.2	20.1
Available K (g kg−1)	24.18	0.159
Specific surface area (m2 g−1)	8.87	--
Average aperture (nm)	16.23	--
Ash content (%)	15.57	--

Total C: total carbon; Total N: total nitrogen; Total P: total phosphorus; Total K: total potassium; Available K: available potassium.

The field-experiment was designed as a two-factor randomized block experiment, including biochar application (B_0_: 0 t ha^−1^ yr^−1^, B_1_: 2.625 t ha^−1^ yr^−1^) and K fertilizer application (K_0_: 0 kg ha^−1^ yr^−1^, K_1_: 60 kg ha^−1^ yr^−1^ K_2_O; potassium sulfate), with totally four treatments (B_0_K_0_, B_0_K_1_, B_1_K_0,_ and B_1_K_1_). All maize straw was collected after harvest and processed into biochar. The air-dried straw was cut into 6-cm segments and carbonized at 500 °C for 2 h in an anoxic vertical kiln (Yang et al., 2022). The biochar application rate was calculated based on the local annual straw yield (7.5 t·hm^−^²) and a 35% carbonization rate, while potassium sulfate was applied at local conventional K fertilizer rates. The basic physicochemical properties of the biochar, tested at the initiation of this long-term experiment in 2013, are presented in **Table 1** (Sun et al., 2021). Three replicated plots with an area of 36 m^2^ (3.6 m × 10 m) were established for each treatment. The experiment was conducted under a continuous monoculture maize system, with maize (cv. Danyu 405, Liaoning Province) sown annually in May and harvested in October. For all treatments, 120 kg ha^−1^ N (urea) and 60 kg ha^−1^ P_2_O_5_ (superphosphate) were applied as base fertilizers each year before sowing. Biochar and chemical fertilizers were incorporated into the soil during the tillage operation, which was performed to a depth of approximately 20 cm. No further fertilizers were top-dressed during the growth period.

### 2.2 Maize plant sampling and analysis

In late September 2021, three uniformly growing maize plants were randomly selected from each experimental plot, carefully uprooted, and transported to the laboratory for further analysis. Fresh plant samples were initially oven-dried at 105 °C for 30 min to deactivate enzymes, followed by drying at 80 °C to constant weight (Wang et al., 2024). The dried samples were then ground to a fine powder and passed through a 0.5-mm sieve. For K analysis, 0.2 g homogenized plant material were digested using the concentrated H_2_SO_4_-H_2_O_2_ method (Bao, 2000), followed by K^+^ determination via flame photometry/atomic absorption spectrometry.

### 2.3 Soil sampling and analysis

The 0–20 cm topsoil samples were collected on 20 October 2021. Five-point samples in each plot were collected to the laboratory, after drying and removing debris, the soil samples were ground and passed through 20 and 200-mesh sieves for subsequent analysis.

#### 2.3.1 Soil mineralogical analysis

Composite soil samples were prepared by homogenizing soil samples from the same treatment. The clay fraction (<2 μm) was separated following the method of Tsao et al. (2013). After repeated treatments with 2–3% hydrochloric acid to remove carbonates, three types of oriented clay mounts were prepared for XRD analysis: air-dried (natural), ethylene glycol-solvated, and heat-treated (550 °C). X-ray diffraction patterns were obtained using a Bruker D8 ADVANCE diffractometer (Billerica, MA, USA) equipped with a graphite monochromator and CuKα radiation (λ = 0.15406 nm). The scanning range was 3–40° (2θ) at a rate of 2° min^−1^ (Das et al., 2019).

#### 2.3.2 Soil apparent K balance calculation

Calculate the soil potassium balance coefficient, potassium surplus, and soil potassium balance based on Equations 1, 2, and 3, respectively. (Hou et al., 2021).

(1) K balance coefficient=Total K input/Total K uptake by crops

(2) K surplus (%)=(Total K input−Total K uptake by crops)/Total K uptake by crops×100

(3) $$\text{Apparent K balance }({\text{kg ha}}^{-1})=\text{Total K input}-\text{Total K uptake by crops}$$

#### 2.3.3 Quantity/intensity relationships

The quantity/intensity (Q/I) curve of soil K was determined according to the method of Das et al. (2019). Briefly, 0.2, 0.3, 0.5, 1.0, 2.0, 3.0, 4.0, 5.0 and 6.0 g soil samples were weighed from each plot, and 50 mL KCl solution (containing 2 mmol L^−^¹ CaCl_2_) with corresponding concentrations of 0, 0.01, 0.05, 0.1, 0.2, 0.5, 1.0, 1.5 and 2.0 mmol L^−^¹ was added sequentially. The mixtures were shaken continuously at 25 °C for 4 h and then equilibrated for 24 h. The concentrations of K^+^, Ca²^+^ and Mg²^+^ in the supernatants were determined using a flame atomic absorption spectrophotometer (AA-7000, Shimadzu, Kyoto, Japan, FAAS).

The Q/I curve parameters are calculated as follows, where Equations 4 and 5 were used to calculate the ΔK value.:

(4) $$\Delta K(mg\;kg^{-1})=(Ci-Cf)\frac{V}{m}$$

(5) $$\Delta K(cmol\;kg^{-1})=\frac{\Delta K(mg\;kg^{-1})}{390}$$

where *ΔK* is the difference in concentration of K^+^ before and after equilibration of the solution; *C_i_* and *C_f_* represent the K^+^ concentration (mg kg^−1^) before and after equilibrium of the solution, respectively; *V* represents the volume (mL) of CaCl_2_ solution added; m is the weight (g) of the soil. The average activity coefficients of the electrolytes K^+^ and Ca^2+^ in the equilibrium solution were calculated by the following Equation 6:

(6) $$lgr\pm =\frac{-Az^{+}z^{-}\sqrt{I}}{1+\alpha \beta \sqrt{I}}$$

where *r±* is the average activity coefficient of the electrolyte; *z^+^* and *z^−^* are the charges of the positive and negative ions, respectively; *I* is the ionic strength of the equilibrium solution; *A* is a temperature-dependent constant of 0.509 at 25 °C. *αβ* are constants related to the ionic radii in the equilibrium solution, with values of 1.9638 for CaCl_2_ and 0.9819 for KCl, respectively. The ionic strength *I* is calculated based on the following Equation 7:

(7) $$I=\frac{1}{2}\sum_{i}C_{i}Z_{i}^{2}$$

*C_i_* is the concentration of *i* ions in the equilibrium solution; *Z_i_* is the valence of the *i* ion. The activity ratio *AR^K^* of K^+^ in the equilibrium solution is based on the following Equation 8:

(8) $$AR^{K}=\frac{C_{K}{\left(r_{KCl}\right)}^{2}}{\sqrt{(C_{Ca}+C_{Mg}){\left(r_{CaCl_{2}}\right)}^{3}}}$$

where *C_K_*, *C_Ca_* and *C_Mg_* are the concentrations of K^+^, Ca^2+,^ and Mg^2+^ in the equilibrium solution, respectively; *r_KCl_* and *r_CaCl2_* are the activity coefficients of KCl and CaCl_2_ in the equilibrium solution, respectively.

The Q/I curve is constructed by taking the activity ratio of K ions (*AR^K^*) in the equilibrium solution as the x-axis and the corresponding ΔK as the y-axis, followed by fitting.

The standard free energy of exchange (−ΔG) for K and Ca/Mg is calculated using the following Equation 9 (Woodruff, 1955):

(9) $$-\Delta \text{G}=\text{RTlnA}R_{e}$$

−Δ*G* represents the energy required for the exchange of equivalent amounts of K^+^ with Ca^2+^ and Mg^2+^ under standard conditions (25 °C). The greater the −ΔG value, the greater the selective adsorption of K^+^ by the soil, which will lead to difficulties in the uptake of K from the soil by the crop, thus predisposing the crop to K deficiency.

#### 2.3.4 Analysis of soil K release kinetics

Soil K release kinetics were determined following the method reported by Das et al. (2021).In brief, 2.00 g of air-dried soil (sieved through a 20-mesh sieve) was weighed into a 50 mL polypropylene centrifuge tube, followed by the addition of 20 mL of 0.005 mol·L^−1^ mixed organic acid solution (citric acid∶ oxalic acid = 1:1, simulating maize root exudates). The mixture was incubated in a thermostatic shaker (25 ± 1 °C, 120 r·min^−1^), with dynamic sampling performed at 1, 5, 10, 24, 48, 72, 96, 120, 150, 216, 312, and 408 h. Prior to each sampling, the mixture was homogenized by shaking at 200 r·min^−1^ for 30 min. Upon completion of incubation, the supernatant was immediately collected by centrifugation at 4000 r·min^−1^ for 10 min, and an equal volume of fresh organic acid mixture was replenished for the next extraction cycle. The K^+^ concentration in the supernatant from each extraction was determined by flame photometry, and the cumulative potassium release curve was fitted using the Elovich kinetic model.

### 2.4 Statistical analysis

The experimental data were systematically compiled using Microsoft Excel 2019 and subsequently analyzed using SPSS Statistics (Version 21.0, IBM Corp., Armonk, NY, USA). A two-way ANOVA was performed, and significant differences were further analyzed by Duncan’s multiple range test at significance levels of 0.05 and 0.01. All figures were generated using Origin 2024 (OriginLab Corporation, Northampton, MA, USA). Soil K release kinetic curves were fitted using the Elovich model. JADE 6.5 (Materials Data, Inc., Livermore, CA, USA) and Origin 2024 software were used for the decomposition of X-ray derivatization images and peak area measurements.

## 3 Results and analysis

### 3.1 Soil clay mineralogy

**Figure 1:**
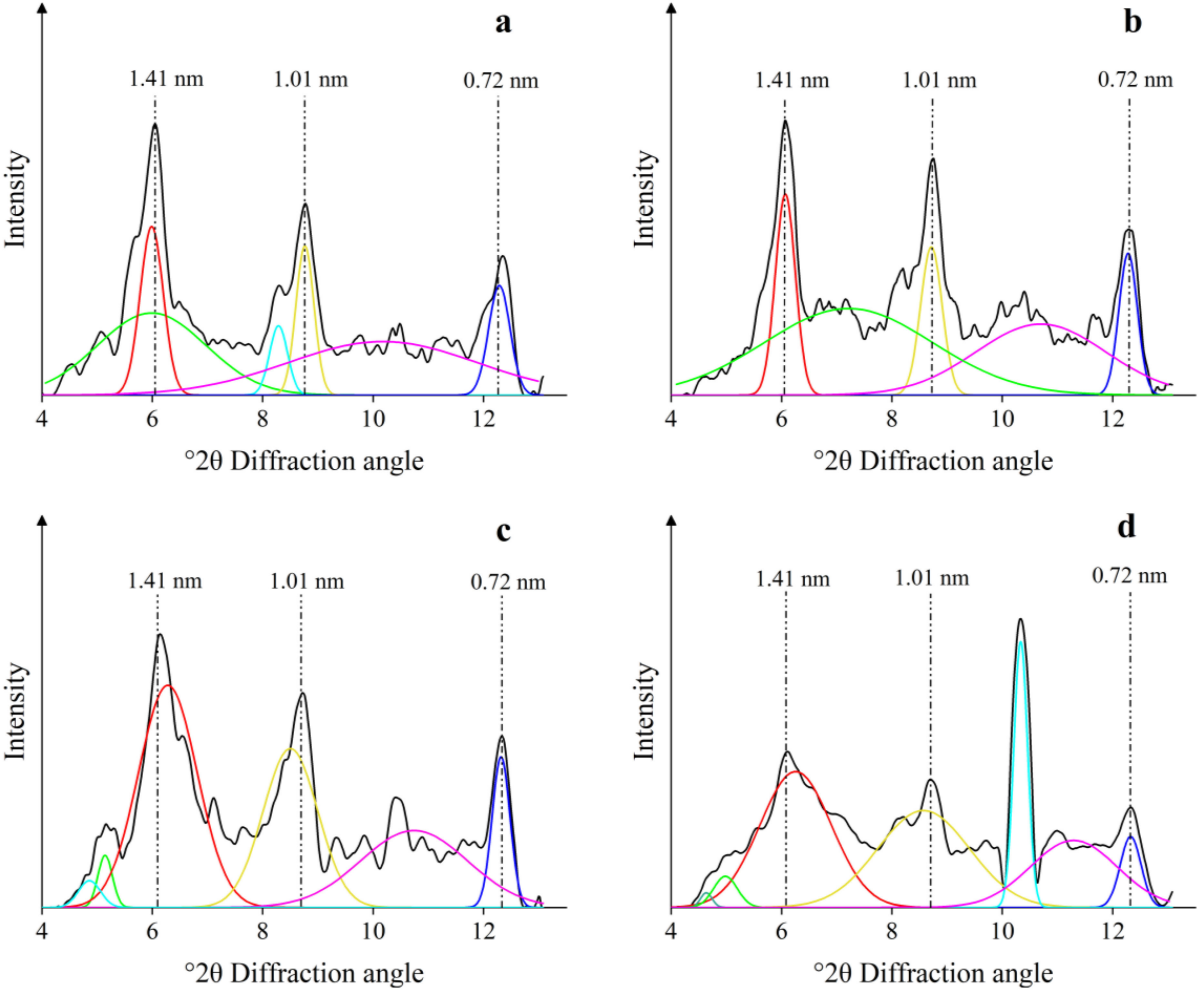
Decomposed X-ray diffraction patterns of clay soil under different treatments [**(a)** B_0_K_0_, **(b)** B_0_K_1_, **(c)** B_1_K_0_, **(d)** B_1_K_1_].

The XRD diffractograms exhibited characteristic broad reflections at d-spacings of 1.41 nm, 1.01 nm, and 0.72 nm (**Figure 1**). Peak deconvolution analysis of the XRD patterns revealed 5–7 constituent peaks per sample, enabling quantitative phase analysis. Based on the positions (2θ), intensities, and shapes of these resolved peaks, the clay mineral composition was systematically classified into seven distinct mineral phases. The d-spacings of 1.41 nm, 1.01 nm, and 0.72 nm correspond to vermiculite, illite, and kaolinite, respectively; peaks with d-spacings ranging from 1.41 to 1.01 nm and from 1.01 to 0.72 nm represent vermiculite/illite interstratified minerals and illite/kaolinite interstratified minerals, respectively.

In the B_0_K_0_ and B_0_K_1_ treatments, soil clay minerals contained mainly vermiculite, vermiculite/illite interlayer minerals, illite, illite/kaolinite interlayer minerals, and kaolinite. Semi-quantitative analysis of XRD patterns showed that compared with the control (B_0_K_0_), the relative contents of vermiculite and kaolinite decreased by 1.49% and 0.30%, respectively, while illite content slightly increased by 0.20% under sole K fertilization (B_0_K_1_). These results indicate that K fertilizer alone exerts a weak overall effect on the main soil clay mineral compositions. In biochar treatments (B_1_K_0_ and B_1_K_1_), clay minerals mainly consisted of vermiculite, illite, illite/kaolinite interlayer minerals, and kaolinite. Biochar amendment facilitated the transformation of vermiculite/illite interlayer minerals to vermiculite and illite. This mineralogical evolution resulted in a significant decrease in the proportion of vermiculite/illite interlayer minerals and an increase in the proportions of vermiculite and illite. Clay mineral compositions differed significantly among treatments (**Table 2**). In the B_1_K_0_ treatment, the relative contents of vermiculite, illite and kaolinite were 38.46%, 25.59% and 7.92%, respectively. Combined application of K fertilizer (B_1_K_1_) further reduced the contents of vermiculite and kaolinite, while illite content continued to increase. This indicates that biochar combined with K fertilizer further promotes the enrichment of illite in clay minerals.

**Table 2:** Soil clay mineral composition of different treatments.

Treatment	Vermiculite (%)	Illite (%)	Kaolinite (%)	Others mineral (%)
B_0_K_0_	13.63	9.44	8.22	68.71
B_0_K_1_	12.14	9.64	7.92	70.30
B_1_K_0_	38.46	25.59	7.92	28.03
B_1_K_1_	31.92	29.28	4.83	33.97

B_0_K_0_: no biochar, no K fertilizer, B_0_K_1_: no biochar, with K fertilizer, B_1_K_0_: with biochar, no K fertilizer, B_1_K_1_: with biochar, with K fertilizer.

Compared to the control (B_0_K_0_), the application of biochar alone (B_1_K_0_) increased the soil illite content by 16.15%, while the combined application of biochar and K fertilizer (B_1_K_1_) further elevated the illite content by 19.84% and simultaneously reduced the kaolinite content by 3.39%. Biochar is the primary driver for elevated soil illite content. Combined with biochar, K fertilizer produces synergistic effects to facilitate illite enrichment and modulate clay mineral assemblages.

### 3.2 Quantity/intensity curves of soil K

**Figure 2:**
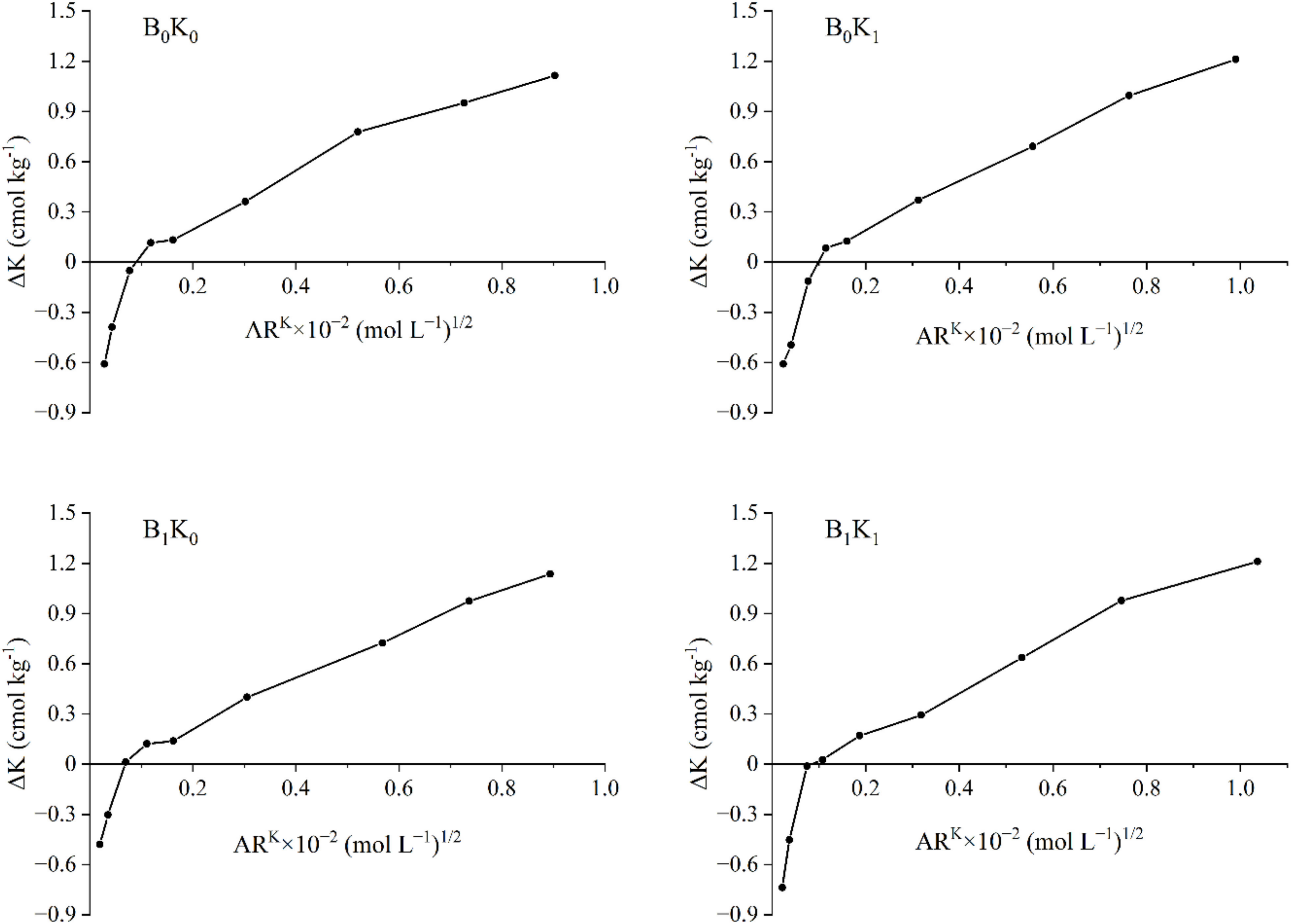
Soil K Q/I curve under different treatments. Data are expressed as mean (*n* = 3). B_0_K_0_: untreated soil; B_0_K_1_: K fertilizer application soil, B_1_K_0_: biochar amended soil, no K fertilizer, B_1_K_1_: biochar amended soil with K fertilizer.

As shown in **Figure 2**, the soil K Q/I curves for all treatments exhibited the typical structural features of standard Q/I profiles: a curved segment at lower AR^k^ on the left and a linear segment at higher AR^k^ on the right. Obvious inflection points were observed in the Q/I curve of each treatment, indicating potassium release from strongly adsorbed sites. While the four Q/I curves shared a similar overall shape, their derived parameters differed significantly.

#### 3.2.1 Soil pool for K supply (−ΔK_0_)

The parameter −ΔK_0_ represents the size of the pool of readily releasable K in the soil (**Table 3**). The −ΔK_0_ differed between treatments, suggesting that long-term application of biochar and K fertilizer affected the soil’s readily releasable K pool. Both biochar and K fertilizer had significant effects on −ΔK_0_, but their interaction did not have a significant effect on −ΔK_0_. Application of biochar resulted in a significant increase in −ΔK_0_ value by 35.70% as compared to no biochar application, application of K fertilizer significantly increased the −ΔK_0_ value by 60.76% as compared to no K fertilizer application. The −ΔK_0_ values for the four treatments in descending order are B_1_K_1_ > B_0_K_1_ > B_1_K_0_ > B_0_K_0_. The −ΔK_0_ values of B_1_K_0_, B_0_K_1_ and B_1_K_1_ treatments were significantly higher by 50.01%, 78.13% and 125.01%, respectively, compared to B_0_K_0_.

**Table 3:** Parameters of soil K Q/I curve under different treatments.

Treatment	Equation	R^2^	−ΔK_0_ (cmol kg^−1^)	PBC (cmol_c_ kg^−1^)	AR_e_ × 10^−3^ (mol L^−1^)^1/2^	K_x_ (cmol kg^−1^)	−ΔG (kJ mol^−1^)
B_0_K_0_	y = 133.83x − 0.032	0.97	0.032 c	133.83 ab	0.24 c	1.15 a	3.33 a
B_0_K_1_	y = 132.10x − 0.057	0.99	0.057 b	132.10 ab	0.43 b	0.90 ab	3.10 bc
B_1_K_0_	y = 135.56x − 0.048	0.99	0.048 b	135.56 a	0.35 b	0.77 b	3.18 b
B_1_K_1_	y = 129.14x − 0.072	0.98	0.072 a	129.14 b	0.55 a	0.95 ab	3.00 c
B	--	--	**	ns	**	ns	**
K	--	--	**	ns	**	ns	**
B × K	--	--	ns	ns	ns	ns	ns

B_0_K_0_: no biochar, no K fertilizer, B_0_K_1_: no biochar, with K fertilizer, B_1_K_0_: with biochar, no K fertilizer, B_1_K_1_: with biochar, with K fertilizer. B: biochar factor, K: K factor, B × K: interaction effect of biochar and K. Data are expressed as mean ± standard deviations (*n* = 3). Different lowercase letters in the same column indicate significant differences among (*p* < 0.05). **is significant at *p* < 0.01 level; -- means not applicable; ns means not significant.

#### 3.2.2 Soil K buffer capacity PBC

Under the condition of biochar application, the PBC value of the K fertilizer treatment was significantly reduced by 4.74% compared to the non-K fertilizer treatment (**Table 3**). Regardless of biochar application, the PBC values of the K fertilizer treatments were lower than those of the non-K fertilizer treatments.

#### 3.2.3 Soil K supply intensity AR_e_

Biochar and K fertilizer applications both had significant effects on AR_e_ values (**Table 3**). Biochar significantly increased AR_e_ by 36.06% compared to no biochar application, and K fertilizer application increased AR_e_ by 65.68% compared to unfertilized soil. The AR_e_ values varied among four treatments, with the B_1_K_1_ treatment showing the highest AR_e_ value of 0.55 × 10^−3^ (mol L^−1^)^1/2^; in contrast, the AR_e_ value in B_0_K_0_ was the lowest of 0.55×10^−3^ (mol L^−1^)^1/2^. The AR_e_ value of B_1_K_1_ was significantly higher compared to B_0_K_0_ treatment by 129.17%.

#### 3.2.4 Soil specific adsorption of potassium K_x_ and standard free exchange energy −ΔG

Long-term application of biochar and K fertilizer resulted in significant differences in K_x_ and −ΔG values (**Table 3**). The soil in B_0_K_0_ treatment had the largest K_x_ and −ΔG values of 1.15 cmol kg^−1^ and 3.33 kJ mol^−1^, respectively. Biochar significantly decreased −ΔG by 4.08% compared to no biochar application, and K fertilizer significantly decreased −ΔG by 6.47% compared to no K fertilizer application. The −ΔG values of the four treatments in descending order were B_0_K_0_ > B_1_K_0_ > B_0_K_1_ > B_1_K_1_, and the −ΔG values of the B_1_K_1_ treatment were significantly decreased by 9.91% as compared to B_0_K_0_.

### 3.3 Soil K release kinetics

**Figure 3:**
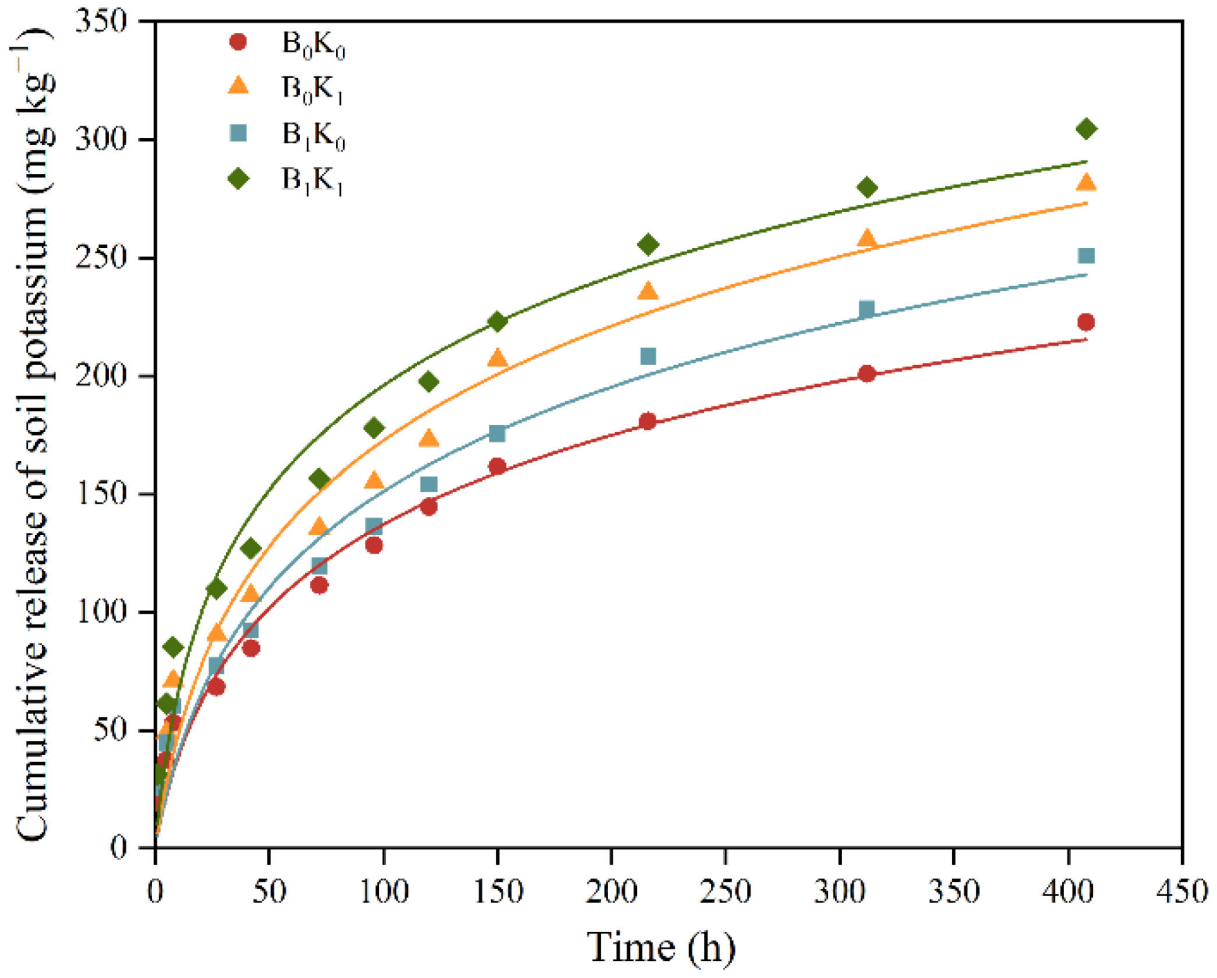
The cumulative release amount of soil potassium over time. Data are expressed as mean (*n* = 3). B_0_K_0_: no biochar, no K fertilizer, B_0_K_1_: no biochar, with K fertilizer, B_1_K_0_: with biochar, no K fertilizer, B_1_K_1_: with biochar, with K fertilizer.

The cumulative release amount of K over time can be divided into a rapid release phase (0–42 h) and a slowly release phase (42–408 h), with the rapid release phase accounting for 36.79–41.68% of the total K release (**Figure 3**). The cumulative release of K varied significantly among the treatments, with B_1_K_1_ showing the highest cumulative release of 304.67 mg kg^−1^, followed by B_0_K_1_, B_1_K_0_ and B_0_K_0_. The cumulative release of K was 26.35%, 12.72% and 36.83% higher in B_0_K_1_, B_1_K_0,_ and B_1_K_1_, respectively, compared to B_0_K_0_. Potassium cumulative release curves were fitted using the Elovich model, and the fitting equations and coefficients of determination R^2^, are shown in **Table 4**. The coefficients of determination R^2^, were all larger than 0.96, indicating a better fit. Among the four treatments, B_1_K_0_ had the highest coefficient of apparent release rate, followed by B_1_K_1_, B_0_K_1,_ and B_0_K_0_ with coefficients of 76.92, 71.43, 71.43, and 58.82, respectively.

**Table 4:** Elovich model fitting for soil K release kinetics.

Treatment	Elovich model	R^2^
B_0_K_0_	y = 58.82 ln (0.092 t + 1)	0.98
B_0_K_1_	y = 71.43 ln (0.075 t + 1)	0.96
B_1_K_0_	y = 76.92 ln (0.087 t + 1)	0.97
B_1_K_1_	y = 71.43 ln (0.153 t + 1)	0.97

B_0_K_0_: no biochar, no K fertilizer, B_0_K_1_: no biochar, with K fertilizer, B_1_K_0_: with biochar, no K fertilizer, B_1_K_1_: with biochar, with K fertilizer.

### 3.4 Maize above-ground dry matter accumulation and K uptake

**Figure 4:**
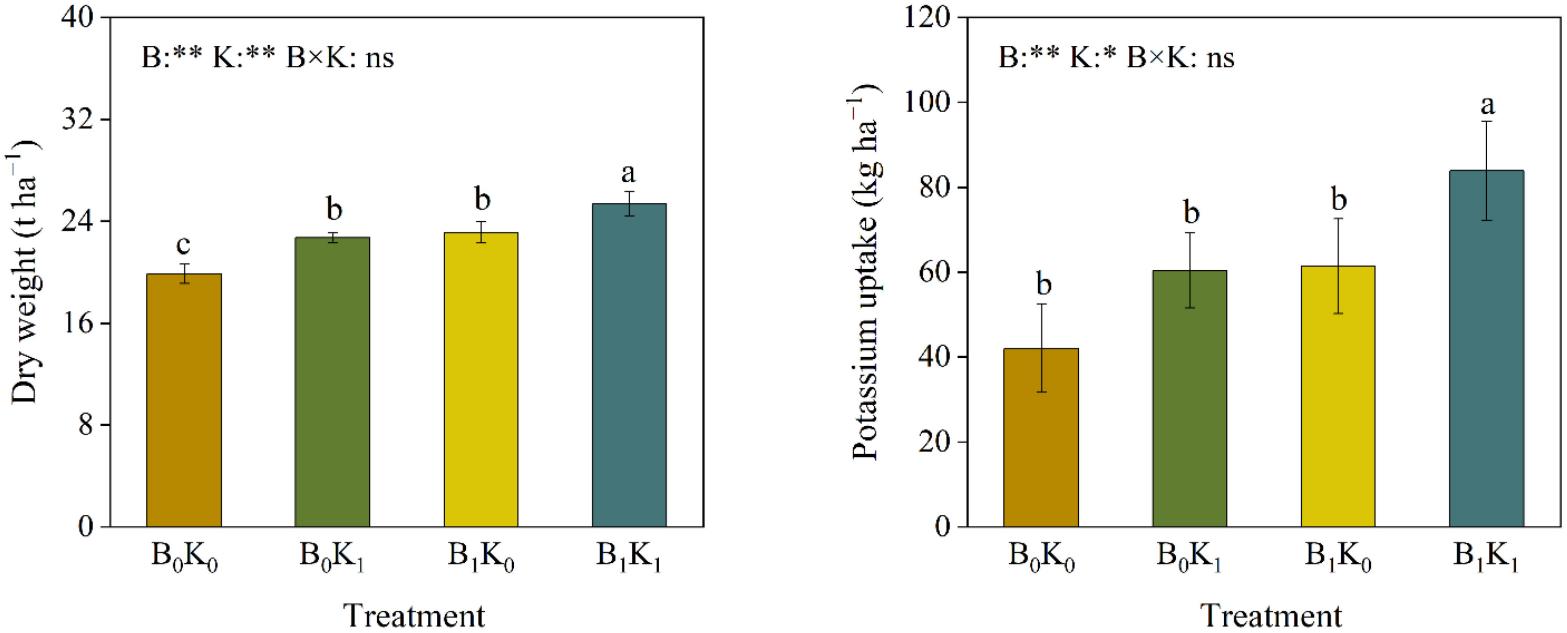
Maize above-ground dry matter accumulation and K uptake under different treatments. B_0_K_0_: no biochar, no K fertilizer, B_0_K_1_: no biochar, with K fertilizer, B_1_K_0_: with biochar, no K fertilizer, B_1_K_1_: with biochar, with K fertilizer. B: biochar factor, K: K factor, B × K: interaction effect of biochar and K. Error bars represent the standard deviations of the mean (*n* = 3). Different lowercase letters indicate a significant difference among the treatments at *p* < 0.05. ** and * indicate significance at *p* < 0.01 and 0.05, “ns” refers to insignificant.

Biochar and K fertilizer application had positive effects on maize biomass accumulation and K uptake (**Figure 4**). Biochar application increased above-ground maize dry weight and K uptake by 13.86% and 41.85%, respectively, compared to no biochar application. Potassium fertilization significantly increased above-ground maize dry weight and K uptake by 11.77% and 39.47%, respectively, compared to no K fertilizer application. Among all the treatments, the B_1_K_1_ treatment had the highest maize dry weight and K uptake, which were significantly higher by 27.59% and 99.51%, respectively, compared to B_0_K_0_.

### 3.5 Soil apparent K balance

The B_0_K_0_ treatment exhibited the most severe soil potassium (K) deficit, showing an apparent K balance of −42.05 kg ha^−1^ (**Table 5**). While the B_0_K_1_ treatment demonstrated some mitigation of this deficit, it still maintained a negative apparent soil K balance. Biochar application alleviated soil K deficiency, converting the K balance from deficit to surplus. The highest K balance (60.63 kg ha^−1^) was observed in the B_1_K_1_ treatment, indicating a synergistic effect between biochar and K fertilizer in enhancing soil K retention.

**Table 5:** Soil apparent K balance under different treatments.

Treatment	Input of K (kg ha^−1^)	K in biochar (kg ha^−1^)	Plant removal of K (kg ha^−1^)	Apparent K balance (kg ha^−1^)	K balance coefficient	K surplus rate (%)
B_0_K_0_	0	0	42.05	−42.05 d	0 c	−100.00 c
B_0_K_1_	60	0	60.41	−0.41 c	1.01 b	0.80 c
B_1_K_0_	0	84.53	61.43	23.10 b	1.40 a	40.50 b
B_1_K_1_	60	84.53	83.90	60.63 a	1.74 a	74.40 a
B				**	**	**
K				**	**	*
B × K				ns	*	*

B_0_K_0_: no biochar, no K fertilizer, B_0_K_1_: no biochar, with K fertilizer, B_1_K_0_: with biochar, no K fertilizer, B_1_K_1_: with biochar, with K fertilizer. B: biochar factor, K: K factor, B × K: interaction effect of biochar and K. Data are expressed as mean ± standard deviations (*n* = 3). Different lowercase letters in the same column indicate significant differences among (*p* < 0.05). ** and * are significant at *p* < 0.01 and *p* < 0.05 levels respectively; ns means not significant.

## 4 Discussion

### 4.1 Effect of long-term biochar application on soil clay mineral composition

The XRD semi-quantitative analysis revealed that 9-years of biochar amendment significantly increased the illite content in the soil. As illite serves as a crucial reservoir of soil K, its increased content can significantly enhance soil K supply capacity (Zhang et al., 2020a). This is because when soil K^+^ concentration is high, K^+^ is preferentially fixed in the interlayers of illite and converted into non-exchangeable K for long-term storage (Lin et al., 2018). When soil solution K^+^ levels decline, illite gradually releases its interlayer K^+^ through a diffusion-controlled process, thereby replenishing plant-available K^+^ in the soil solution (Sparks and Liebhardt, 1981; Lin et al., 2018). In this study, biochar increased soil illite content mainly through two mechanisms: (1) Biochar application directly replenishes the soil K pool through the release of K^+^ (Xiu et al., 2023). This process markedly increases soil available potassium content (Bao et al., 2024a) and maintains a positive potassium balance within the soil system. By alleviating soil K deficiency, this approach reduces the reliance of crops on interlayer K release from illite, thereby protecting the crystalline structure of illite from degradation (Darunsontaya et al., 2010). (2) Cycling of mineral nutrients and related biochemical processes are strongly regulated by soil microbial activity (Zhang et al., 2022). Biochar application restructures soil microbial communities and suppresses their degradative effects on clay minerals, thereby effectively promoting the accumulation of illite in soils (Zhang et al., 2020a).

### 4.2 Effect of long-term biochar application on Q/I curve parameters

In this study, the order of soil K supply pool (−ΔK_0_) under different treatments was B_1_K_1_ > B_0_K_1_ > B_1_K_0_ > B_0_K_0_. This variation primarily stems from differences in the ability of each treatment to replenish and maintain soil K pools. Specifically, the B_0_K_0_ treatment, which lacked long-term exogenous K input, likely led to continuous depletion of active K from the interlayer and surface of clay minerals (Das et al., 2019), resulting in the lowest −ΔK_0_ value. In comparison, the B_1_K_1_ treatment not only supplied K for crop growth through conventional fertilization but also benefited from biochar addition: biochar itself is rich in readily available K for crops (Xiu et al., 2023), and its unique pore structure and surface properties effectively retain K^+^ and prevent its migration to deeper soil layers (Liu et al., 2019), thus resulting in dual benefits. This synergistic effect significantly enhanced the −ΔK_0_ value.

Generally, it is recognized that higher potential buffering capacity (PBC) values indicate greater soil capacity to maintain solution K^+^ concentrations (Wei et al., 2024). However, our results revealed that the PBC values of K fertilization treatments (B_0_K_1_ and B_1_K_1_) were lower than those of non-K treatments. This phenomenon may be attributed to long-term application of K fertilizers altering the properties of soil colloids, thereby enhancing the soil’s capacity to fix K. This process shifts more K from the “non-specifically adsorbed” pool into the more stable “specifically adsorbed pool.” When continuous long-term cultivation gradually depletes non-specifically adsorbed K^+^, the specifically adsorbed pool releases K^+^ to maintain the K^+^ concentration in the soil solution (Abaslou and Abtahi, 2008; Zhang et al., 2011; Islam et al., 2017). The AR_e_ values of all treatments were below the critical level of 1×10^−3^ (mol L^−1^)^1/2^ (Beckett, 1972), indicating that the readily available K in the tested soil primarily originates from specific wedge-shaped sites and edge sites (Xiu et al., 2025).

Long-term application of biochar or K fertilizer significantly reduced soil −ΔG values, with the B_1_K_1_ treatment showing the lowest −ΔG value. In contrast, the B_0_K_0_ treatment (3.3 kJ mol^−1^) approached the critical threshold for severe K deficiency (≥3.5 kJ mol^−1^). These results demonstrate that the combined application of biochar and K fertilizer effectively reduced −ΔG values, thereby weakening the soil selective adsorption of K^+^ (Roy et al., 1991) and improving K bioavailability. This improvement was specifically manifested through the synergistic effects of: an enhanced K supply pool (−ΔK_0_), reduced K release free energy (−ΔG), and an optimal K supply intensity (AR_e_). Together, these factors significantly increased soil K availability, further promoted crop K uptake and utilization, leading to substantial increases in crop dry weight, K absorption.

### 4.3 Effect of long-term biochar application on soil K release kinetics

A continuous extraction method using a low-concentration oxalic-citric acid mixed solution to simulate the dissolution process of K-bearing minerals by low-molecular-weight organic acids secreted from maize roots was employed in this study (Silva et al., 2008). The results indicated that after 9 years of biochar amendment, there were significant differences in cumulative K release among the different treatments. The B_1_K_1_ treatment demonstrated the highest cumulative K^+^ release (304.67 mg kg^−1^), likely resulting from: (1) the combined application of biochar and K fertilizer contributed to a persistent K surplus in the soil under this treatment. Meanwhile, the high porosity and large specific surface area of biochar improved K^+^ adsorption and exchange capacity, which effectively reduced K leaching and prolonged its availability, leading to a significant increase in the contents of various K forms (Bao et al., 2024a; 2024b); (2) long-term application of biochar promoted the accumulation of illite minerals in the soil, resulting in its highest relative content among all treatments (**Table 2**). Studies have shown a positive correlation between illite content and soil K release (Zhang et al., 2020a), (3) most favorable thermodynamic parameters as evidenced by the largest −ΔK_0_ and smallest -ΔG values. The synergistic effects of these multiple factors collectively shaped the biphasic kinetic pattern of soil K^+^ release: (1) an initial rapid release phase (0–72 h), predominantly governed by the dissolution of water-soluble K^+^ and the desorption of exchangeable K^+^ from mineral edges (Xiong et al., 2023); and (2) a subsequent slow release phase (72–408 h), likely sustained by K^+^ diffusion from planar and interlayer sites of clay minerals (Das et al., 2021).

Long-term experiments offer greater advantages over short-term studies in distinguishing the effects of different treatments on soil K supply capacity, as they can clearly reflect fertility differences among treatments through cumulative effects. Insufficient K inputs over time will gradually deplete soil K reserves, whereas this study confirms that the continuous long-term application of straw-derived biochar and K fertilizer can directly supplement K and enhance its retention capacity, thereby improving the overall K supply level of the soil (Zhu et al., 2021).

## 5 Conclusions

This study elucidated the mechanisms underlying the effects of 9 consecutive years of biochar application on soil K supply capacity and K accumulation in maize plants. The results showed that biochar increased soil illite content, expanded the K supply pool (−ΔK_0_) and raised K activity ratio (AR_e_), while reducing the free exchange potential of soil K (−ΔG). These combined effects markedly improved soil K supply capacity. Such changes facilitated K uptake by maize plants and significantly increased plant K accumulation. Meanwhile, biochar promoted the continuous release of soil KK and effectively alleviated the negative K balance in soil. The findings provide a scientific basis for applying biochar to improve soil K supply capacity and availability, and to achieve sustainable maize production. However, this experiment was conducted only under a single soil type and cropping system. Further validation is required across diverse regions, crop species and biochar types in future studies.

## Data Availability

The raw data supporting the conclusions of this article will be made available by the authors, without undue reservation.
